# Actual facts about bacterial meningitis

**DOI:** 10.1186/1471-2334-14-S7-O36

**Published:** 2014-10-15

**Authors:** Narcisa Nicolescu, Alexandru Crişan, Emilia Nicoară, Voichița Lăzureanu, Virgil Musta, Ruxandra Laza, Roxana Nicolescu

**Affiliations:** 1 Dr. Victor Babeş University of Medicine and Pharmacy, Timişoara, Romania

## Background

Bacterial meningitis still represents an important chapter in the present infectious pathology, despite the increasing progress in the prophylaxis and prevention measures. Apart from tuberculous etiology, and of course the well-known classical etiologies – meningococcal, pneumococcal, *Haemophilus influenzae*, staphylococcal and Gram-negative bacteria – new entities that need to be evaluated seem to appear.

Objectives: 1. To determine the incidence of bacterial meningitis in the last years, in comparison with the international situation encountered in this pathology. 2. To detect new predisposing and risk factors for meningitis. 3. The important role of procalcitonin and other markers for the quick diagnosis of meningitis. 4. The utilization in the current medical practice of modern molecular diagnostic tests, especially for the severe and atypical cases.

## Methods

We have studied and evaluated in a retrospective study 87 cases of bacterial meningitis, which were hospitalized in the Infectious Disease Hospital “Victor Babeş” Clinic II during the period 2011-2013. These cases were analyzed based on different criteria: age, sex, pathological antecedents, modalities of disease onset, clinical state at admission to hospital, biochemical characteristics of the cerebrospinal fluid (CSF), bacteriological diagnostic, paraclinical investigations, imaging, treatment and disease evolution.

## Results and conclusion

The clinical presentation of infectious diseases, in particular meningitis, can be very atypical, a fact that creates significant diagnostic problems during the therapeutic time window. In our study the most common etiologies, meningococcal, pneumococcal and *Haemophilus influenzae*, were encountered in almost equal proportions. Lactic acid elevation and decrease of glucose level in CSF are still very useful for the diagnosis of bacterial meningitis. With the help of the medical microbiologist and blood culture machine Vides, doctors had the opportunity to discover new bacterial etiologies such as: *Granulicatella adiacens*.

